# Severe Leptospirosis: A Case Report

**DOI:** 10.7759/cureus.30712

**Published:** 2022-10-26

**Authors:** João Cardoso, Ana Gaspar, Cristina Esteves

**Affiliations:** 1 Internal Medicine, Hospital de Santarém, Santarém, PRT

**Keywords:** acute kidney disease, pulmonary hemorrhage, rats, poor sanitation, zoonotic infection, leptospirosis

## Abstract

Leptospirosis is an infection caused by *Leptospira*. Leptospirosis causes disease in humans mainly in developing countries and also in countries with poor housing and sanitation, due to animals (mainly rats) that are potential sources of contamination. The clinical manifestations and the severity of leptospirosis are highly variable.

We present the case of a 56-year-old female that was admitted to the emergency department with a three-week history of fever (38.4ºC), headache, dyspnea, and cough. There was a worsening of the patient's clinical status with respiratory failure and the necessity of admission to the intensive care unit for respiratory support with mechanical ventilation. The treatment was initiated with piperacillin and tazobactam, azithromycin, and steroids. There was a favorable evolution, and the patient was transferred to the internal medicine ward after 12 days with a suspected diagnosis of small vessel vasculitis and pneumonia. In the medical ward, after a careful anamnesis, leptospirosis was suspected and confirmed.

The aim of this case report is to highlight the importance of a good anamnesis and the fact that an elaborate clinical history helps to consider new diagnostic hypotheses. Also intends to alert to the existence of leptospirosis in developed countries, a disease underdiagnosed in these countries.

## Introduction

Leptospirosis is a zoonosis caused by the spirochete *leptospira*. The incubation period is usually seven to 13 days but may vary from day 2 to day 20 [1]. The incidence of Leptospirosis is increasing globally on a large scale, even in developed countries, and its transmission and clinical presentation have been varying according to socioeconomic conditions and environments. It is more frequent in low-income countries of tropical regions and in developed countries leptospirosis has been neglected [2].

In 2018, in Portugal, it was confirmed 69 cases of Leptospirosis, a rate of 6.7 cases per million inhabitants, and in Europe, the incident has been reported to be 1.6 cases per million inhabitants [3]. According to the latest statistical data, the number of cases appears to be increasing in Europe [4].

The most common animals that play an important role in the transmission of human leptospirosis are rodents, in particular rats [5]. The infection is acquired by direct contact with infected urine and also by indirect exposure or contact with contaminated water or soil. Infection by rat urine is the more frequent form of infection [6].

The clinical presentation of Leptospirosis is unclear and can vary from flu-like febrile illness to rapidly fatal infection. Typically present with fever, headache, and myalgia but symptoms of any organ may be apparent [7]. Severe forms, like meningitis, pulmonary hemorrhage, Weil's syndrome with jaundice, and acute kidney injury, are presented in only 10% of reported cases [6].

The differential diagnosis is difficult due to the overlap of its clinical presentations that can mimic other infectious (dengue, influenza, malaria, enteric fever, toxoplasmosis, hepatitis, etc.) or auto-immune diseases [2]. Leptospirosis can be diagnosed by cultures, serological methods, or, more recently, by molecular techniques. Leptospiremia occurs before the onset of symptoms, and it ends after the first week of illness. Antibodies are detectable in the blood approximately five to seven days after the onset of symptoms and definitive serological investigation in leptospirosis remains the microscopic agglutination test (MAT), the principal test recommended and the standard gold test for diagnosis [8]. A MAT result is considered positive when titers are > 100 [4]. In an analysis, it was concluded that the culture was positive in 48% of the cases, the PCR in 62%, and the serology in 97% of the confirmed cases [8].

The majority of leptospirosis cases are mild and will resolve spontaneously. However, treatment with antibiotics must be initiated as soon as possible to avoid complications and, in some patients, the progression of the disease to more severe forms can be prevented by early initiation of antibiotic therapy. Mild cases can be treated with oral doxycycline, amoxicillin, or azithromycin. In severe cases should be used intravenous penicillin, cefotaxime, or ceftriaxone [2].

## Case presentation

A 56-year-old female patient with a personal history of peptic ulcers, obesity, and active smoking. She was admitted to the emergency department with a three-week history of fever (38.4ºC), headache, dyspnea, and cough.

**Figure 1:**
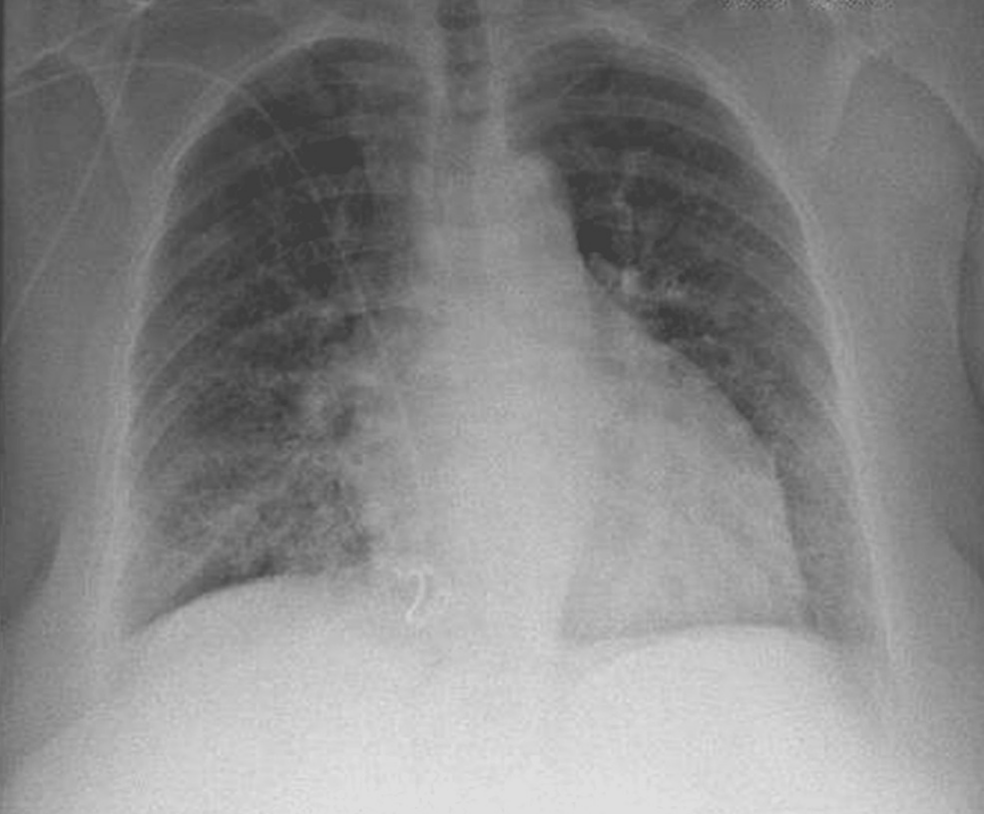
Admission chest x-ray

**Figure 2:**
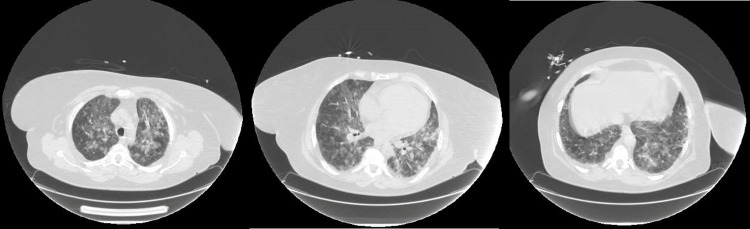
Chest CT scan, axial planes

Laboratory tests revealed hemoglobin level of 10.5 g/dL, C-reactive protein level of 24.3 mg/dL, and creatinine level of 5.3 mg/dL. Hepatic and coagulation function values were within normal parameters. Gasometry revealed respiratory insufficiency type 1 and metabolic acidemia. Image exams were performed chest x-ray (Figure 1) showed a diffuse infiltrative opacification and the chest computed tomography scan (Figure 2) revealed diffuse multifocal bilateral ground-glass densification in all lung lobes, with some areas with a tendency to coalesce, aspects that were compatible with the diagnostic hypothesis of alveolar hemorrhage or acute respiratory distress syndrome. Blood, sputum, and urine cultures were performed and later came back negative.

Antibiotic empiric treatment was initiated with piperacillin and tazobactam and azithromycin for suspected pneumonia. It was also suspected small vessel vasculitis (pulmonary-renal syndrome), so steroids (methylprednisolone) were started. 

On the first day of hospitalization, due to worsening symptoms, she was admitted to the Intensive Care Unit (ICU) with the suspected diagnosis of Community Acquired Pneumonia with respiratory failure requiring invasive mechanical ventilation and small vessel vasculitis (alveolar hemorrhage and acute kidney injury.

In the ICU, a transthoracic echocardiogram was performed, which revealed good systolic and diastolic function and the absence of regurgitation or stenosis. A bronchoscopy was also performed, which revealed the presence of blood distributed throughout the bronchial tree, coming mainly from the upper lobar bronchi, compatible with alveolar hemorrhage.

By day 13, due to clinical improvement, she was transferred to the internal medicine ward. The autoimmune study performed was normal. However, as it showed improvement in the treatment performed, the ICU discharge diagnosis remained small vessel vasculitis and pneumonia. After a careful anamnesis, leptospirosis was suspected because she worked in a prison kitchen with poor hygiene conditions and sometimes rats were seen. It was requested MAT for *leptospira* and the diagnosis was confirmed.

The main complication during hospitalization was critical illness myopathy and physiotherapy was performed with good response. As intercurrence also stands out as acute cystitis to Enterobacter Aerogenes (isolation in the bacteriological examination of urine). She underwent six days of antibiotherapy with Cefuroxime.

She was discharged from the hospital for follow-up in Internal Medicine and Rehabilitation Physical Medicine. With the articulation of the Hospital Physiotherapy Service and the outpatient Physiotherapy Teams, the patient underwent physiotherapy at the hospital twice a week and at home three times a week. At the internal medicine appointment, complementary exams were performed, such as analyses, chest x-ray and echocardiograms, to assess her recovery. After three months, the patient returned to work feeling well.

**Table 1:** Investigations on admission and days later

	Value				Reference Range
	Day 1	Day 13	Day 16	Day 24	
White blood cells	8.8 x 10⁹/L	10.0 x 10⁹/L		7.6 X 10⁹/L	4.0-10.0
Neutrophils	7.7 x 10⁹/L	8.6 x 10⁹/L		6.2 X 10⁹/L	2.0-7.0
Lymphocytes	0.8 x 10⁹/L	1.0 x 10⁹/L		1.0 X 10⁹/L	1.0-3.0
Hemoglobin	10,5 g/dL	8.3 g/dL		10.2 g/dL	12.0-15.0
C-reactive proteins	24,30 mg/dL	1.09 mg/dL		2.31 mg/dL	<0.50
Creatinine	5.3 mg/dL	0.7 mg/dL		0.7 mg/dL	0.6-1.1
Serum bilirubin	0.0 mg/dL	0.6 mg/dL		0.6 mg/dL	0.2-1.2
pH	7.332	7.395		7.390	7.350-7.450
pO2	113.0 mmHg	113.0 mmHg		92.3 mmHg	83.0-108.0
pCO2	37.9 mmHg	45.6 mmHg		43.0 mmHg	32.0-48.0
HCO3 −	20.0 mmol/L	26.8 mmol/L		25.1 mmol/L	21.8-26.2
Lactate	1.1 mmol/L	1.8 mmol/L		1.5 mmol/L	0.5-1.6
Leptospira		>100 Positive			
Blood Cultures	Negative	Negative	Negative	---------------	
Urine Culture	Negative	Negative	Enterobacter Aerogenes	---------------	

## Discussion

Leptospirosis is a zoonosis with a 10 times higher incidence in the tropics than in temperate regions [6]. It is caused by *Leptospira,* a pathogenic spirochete, and human infection occurs after exposure to environmental sources, mainly animal urine, contaminated water or soil, or infected animal tissue [6]. Portals of entry include abrasions or cuts in the mucous membrane [2]. Humans at risk of contracting Leptospirosis are people living in poor and unsanitary housing, campers, farmers, and travelers [4].

Our patient worked in a place with poor sanitary conditions, as the prison kitchen was located in a very old building, with dirty drains without good maintenance, and rats were often seen in the pantry and sometimes on the kitchen floor. The infection occurred during the summer (August) and the patient mentioned wearing sandals at work. In Europe, Leptospirosis is a seasonal disease and the majority of cases occurred between July and October (maximum incidence in August and September) [4].

It is a disease usually reported in low-income countries of tropical regions and in developed countries travel related leptospirosis is considered a significant source of infection [2]. In 2018, there were 69 cases of Leptospirosis in Portugal, less than seven cases per million, and in Europe, the incidence reported was 1.6 cases per million inhabitants [4]. It is an unusual disease in Portugal and even less so in Europe, where it remains as a rare disease [4]. In Europe, has notably been associated with urban transmission, a phenomenon still not fully understood [4]. Although it has been increasing in developed countries, leptospirosis has been regarded as a neglected disease [2]. If there is no knowledge of its existence or if the physicians are not aware of it, it will be treated as another disease or even worse, not treated. In the case of our patient, her treatment approach was for pneumonia and autoimmune disease, small vessel vasculitis.

The clinical manifestations and the severity of leptospirosis are highly variable and the lack of pathognomonic presentation for leptospirosis makes a final diagnostic dependent on serologic tests [9]. Confirmatory tests are slow, the disease can progress rapidly, and a delay in diagnosis can be fatal [6].

Empirical antibiotic treatment should be initiated once a suspected leptospirosis is made [6]. For mild cases, it can be used doxycycline 100 mg orally twice a day or amoxicillin 500 mg QID, or azithromycin 500 mg once daily for three days [2]. For severe cases, treatment could be ampicillin at a dose of 0.5-1g IV q6h, penicillin 1.5 million units IV four times a day, ceftriaxone 1g IV once a day, or cefotaxime 1g IV QID for seven days [2].

## Conclusions

Leptospirosis is a common disease in tropical countries but considers a rare disease in European countries. It has a variable clinic that is easily confused with other diagnoses. If physicians are not aware of its existence or if the anamnesis is incomplete and does not include professional or occupational habits, patients will be mistreated.

High suspicion is important to consider leptospirosis as a diagnostic hypothesis because in its severe form, as happened to our patient, it can progress with fast clinical deterioration. Empirical treatment should be started as soon as there is a suspicion of leptospirosis.
